# The role of exercise parameters on small extracellular vesicles and microRNAs cargo in preventing neurodegenerative diseases

**DOI:** 10.3389/fphys.2023.1241010

**Published:** 2023-08-15

**Authors:** Francesco Fischetti, Luca Poli, Marina De Tommaso, Damiano Paolicelli, Gianpiero Greco, Stefania Cataldi

**Affiliations:** ^1^ Department of Translational Biomedicine and Neuroscience (DiBraiN), University of Study of Bari, Bari, Italy; ^2^ Applied Neurophysiology and Pain Unit, Department of Translational Biomedicine and Neuroscience (DiBraiN), Policlinico General Hospital, University of Study of Bari, Bari, Italy; ^3^ Neurophysiology Operative Unit, Department of Translational Biomedicine and Neuroscience (DiBraiN), Policlinico General Hospital, University of Study of Bari, Bari, Italy

**Keywords:** physical activity, sport, exosome, microvesicles, Parkinson, Alzheimer, ALS, neuroprotection

## Abstract

Physical activity (PA), which includes exercise, can reduce the risk of developing various non-communicable diseases, including neurodegenerative diseases (NDs), and mitigate their adverse effects. However, the mechanisms underlying this ability are not yet fully understood. Among several possible mechanisms proposed, such as the stimulation of brain-derived neurotrophic factor (BDNF), endothelial nitric oxide synthase (eNOS), insulin-like growth factor-1 (IGF-1), vascular endothelial growth factor (VEGF), and nerve growth factor (NGF), the possible involvement of particular vesicular structures enclosed in lipid membranes known as extracellular vesicles (EVs) has recently been investigated. These EVs would appear to exert a paracrine and systemic action through their ability to carry various molecules, particularly so-called microRNAs (miRNAs), performing a function as mediators of intercellular communication. Interestingly, EVs and miRNAs are differentially expressed following PA, but evidence on how different exercise parameters may differentially affect EVs and the miRNAs they carry is still scarce. In this review we summarized the current human findings on the effects of PA and different exercise parameters exerted on EVs and their cargo, focusing on miRNAs molecules, and discussing how this may represent one of the biological mechanisms through which exercise contributes to preventing and slowing NDs.

## 1 Introduction

Neurodegenerative diseases (NDs) are characterized by progressive nerve cell deterioration, leading to neurodegeneration and disability (Dugger and Dickson, 2017). Although NDs represent a serious health problem in communities, modifiable risk factors, such as physical activity (PA), have received relatively little attention. These disorders are becoming more prevalent, in part because the elderly population has increased in recent years (Heemels, 2016). The most frequent NDs are dementias such as Alzheimer’s disease (AD) and frontotemporal dementia (FTD), which account for 60%–70%. Other NDs include Parkinson’s disease (PD), which is the second most frequent ND, Huntington’s disease (HD), motor neuron diseases such as amyotrophic lateral sclerosis (ALS), multiple sclerosis (MS) and spino-cerebellar ataxias. These diseases differ in their pathophysiology: some cause impairments in memory and cognition, while others affect the ability to move, speak, and breathe (Abeliovich and Gitler, 2016; Canter et al., 2016; Taylor et al., 2016; Wyss-Coray, 2016; Filippi et al., 2018). There is currently no cure for any of these diseases, though not for lack of trying (Heemels, 2016).

Epidemiological studies have shown that PA can reduce the risk of AD and dementia by 45% and 28%, respectively (Hamer and Chida, 2009). In recent years, PA has shown its potential as a therapeutic approach that can modify NDs (Frederiksen et al., 2018). PA has been described as a nondrug therapy against numerous diseases such as neurological, metabolic, psychiatric and cardiovascular diseases (Pedersen and Saltin, 2015).

With the great advancement of molecular techniques, researchers have identified several molecules whose production is found to be stimulated by PA, such as increased superoxide dismutase (SOD) (Feter et al., 2019), brain-derived neurotrophic factor (BDNF) (Erickson et al., 2011; Coelho et al., 2014), endothelial nitric oxide synthase (eNOS) (Lange-Asschenfeldt, and Kojda, 2008), insulin-like growth factor-1 (IGF-1), vascular endothelial growth factor (VEGF) (Maass et al., 2016), and nerve growth factor (NGF), and decreased production of damaging free radicals in the hippocampus region of the brain, which are mainly involved in memory (Ionescu-Tucker and Cotman, 2021). However, the exact mechanism by which PA exerts these effects is not yet fully understood. Understanding the molecular and cellular mechanisms of exercise-induced benefits is of great interest for preventive and therapeutic implications.

Recently, it has been observed that, during exercise, various organs and cells communicate through the secretion of biomolecules called exerkines. These molecules, including myokines, adipokines, hepatokines, act as signaling molecules that can have autocrine, paracrine, and endocrine effects. They are released into circulation and can influence multiple tissues and organs throughout the body. Exerkines play a crucial role in mediating the beneficial effects of exercise on various physiological processes, including muscle mass, metabolism, immune function, cardiovascular health, and neuroprotection (Magliulo et al., 2022; Rody et al., 2022). To preserve their integrity during transit, these molecules need to be transported by specialized structures such as extracellular vesicles (EVs), a wide family of membranous structures, including the most well-known exosomes (EXs) (Théry et al., 2018; van Niel et al., 2018), which facilitate intercellular communication and cross-talk between different organs (Magliulo et al., 2022). Consequently, EVs and specifically EXs have been proposed as an additional mechanism, probably underlying others already identified, through which PA may exert preventive and therapeutic effects on different kinds of pathologic conditions, including NDs (Whitham and Febbraio, 2019; Rigamonti et al., 2020). However, it is still unclear how exercise parameters like frequency, intensity, time, type, volume, and progression (FITT-VP) can affect the modulation of EVs and their cargo. There is still a gap between precisely characterizing the molecular pathways that exercise induces and the possible health advantages that have been reported. Developing tailored exercise interventions and creating molecular-level interventions that mimic exercise could benefit from a deeper understanding of these biological processes and pathways (Sanford et al., 2020). The study of EVs is still in its infancy, especially in terms of its relationship to PA. Therefore, we explore and summarize the currently available human evidence, regarding the effects that PA and related FITT-VP parameters may exert on EVs and their cargo, focusing on miRNAs molecules, aiming to examine how this process may represent one of the biological mechanisms by which exercise plays a role in the prevention and slowing of NDs.

## 2 Extracellular vesicles

EVs, defined as eukaryotic cell membrane fragments, consist of a phospholipid bilayer and serve as carriers of various molecules (DNA, mRNA, microRNA, cytokines, proteins and lipids), protecting them from degradative agents during transport in the bloodstream (Szatanek et al., 2017). Based on their origin and size, EVs are mainly divided into exosomes, microvesicles, and apoptotic bodies. Exosomes (50/150 nm ø) are EVs that originate from the late endosome, while microvesicles (100/1,000 nm ø) originate directly from the plasma membrane and apoptotic bodies (100/5,000 nm ø) are released following cell fragmentation during the late phase of apoptosis (Yuan et al., 2019).

Cells of the central nervous system (CNS), like neurons, oligodendrocytes and astrocytes, also secrete EVs, important in intracellular communication, neuronal plasticity and myelination, as well as in neuroprotective and regenerative processes. EXs appear capable of carrying enzymes with metabolic functions (e.g., catalase and superoxide dismutase-1) that are useful to neurons for increased resistance to oxidative stress (Yuan et al., 2019). However, the role played by EVs in the CNS remains a subject of conflicting evidence. Trotta et al. (2018) highlight their potential involvement in NDs like AD, PD, HD and ALS. These EVs are believed to facilitate the transport of misfolded proteins (tau, amyloid-β, and α-synuclein), which are implicated in the pathogenic process. The authors suggest that under normal conditions, glial cells secrete trophic and protective factors for neurons, but in pathological conditions, they undergo a phenotypic change, contributing to the spread of toxicity through EVs. Similarly, D’Anca et al. (2021) report that in MS, the first neurological disease in which EVs have been detected, it appears that these vesicles may play a role in myelin damage and inflammation but, on the other hand, they could also exert a neuroprotective role, being involved in the processes of modulating synaptic plasticity in the CNS and repairing damaged neurons.

Recent studies have suggested that EVs like EXs play a role in organ-to-organ communication, moreover, have shown that exercise can lead to an increase in the release of EVs, into the bloodstream. In addition, exercise can stimulate EXs production and microRNAs (miRNAs) processing in muscle, which may have implications for systemic metabolism. Furthermore, circulating exosomal miRNAs (exomiRs) have been found to be altered by acute exercise, with specific miRNAs showing changes in expression levels. These exercise-regulated exomiRs often target components of the IGF1 signaling pathway, which is involved in muscle growth and glucose homeostasis. Additionally, EVs secreted during exercise originate from various cell types in the circulatory system, suggesting a heterogeneous pool of cells involved in regulating systemic metabolism. The molecular mechanisms underlying EVs biogenesis and release are still not fully understood, but intracellular calcium release and signaling have been implicated. The release of EVs may be accelerated in muscle due to the large flux in calcium during muscle contractions (Sabaratnam et al., 2022).

EXs, currently, are the most studied EVs, although terminological confusion exists. Unless the EV is captured in the act of release with live imaging techniques, assigning an EV to a particular biogenesis pathway remains difficult, as a consensus on EV subtype-specific markers has not yet emerged (Théry et al., 2018). Because of the challenges to distinguishing between different classes of EVs with current isolation techniques, the term exosome-like EVs (ELVs) appears to be an appropriate term of use that encompasses the smallest EVs (exosomes and small micro-vesicles) (Warnier et al., 2022). The studies’ main characteristics and findings are shown in Table 1.

**TABLE 1:** Summary characteristics of reviewed studies.

			Exercise intervention						
Authors	Sample size	Subjects age (years)	Frequency	Intensity	Time	Type	Volume/Progression	Extracellular vesicles-used terminology	Main outcomes
Frühbeis et al. (2015)	*n* = 12 (M) CyG (*n* = 8) TmG (*n* = 4)	CyG: 41.1 ± (14.9)	Single bout	CyG: 50w increasing by 50w every 3′, until exhaustion		Acute aerobic exercise		EVs	CyG: ↑ 2.7-fold ELV particles count post-ex. ↓ to baseline after 90′
		TmG: 27 ± (2.1)		TmG: 6 km/h increasing by 2 km/h every 3′, constant grade of 1.5%, until exhaustion					TmG: ↑ 1.5-fold ELV particles count post-ex. ↔ stayed elevated up to 24 h post-ex
Guescini et al. (2015)	*n* = 18 (M) (BMI: 22.7 ± 2.9)	26 ± (4.8)	Single bout	AAE: 80% of VO_2max_	AAE: 40′	Graded running test/Acute aerobic exercise		EVs	↑ ELV-miRNAs (miR181a-5p, miR133b, miR206)
Aoi et al. (2013)	*n* = 11 (M)	21.5 ± (4.5)	Single bout	70% VO_2max_	Acute: 60′	Acute aerobic cycling exercise			↔ (miR-1, miR-133a, miR-133b, miR-206, miR-208b, miR-499)
			+		Chronic: 30′	+			↓ miR-486 post-ex
			3x weeks/4 weeks			Chronic aerobic exercise			
D’Souza et al. (2018)	*n* = 10 (M) (BMI: 24.1 ± 2.1)	24.6 ± (4.0)	Single bout	peak power output		High-Intensity Interval exercise	10 sets x 60″ (75″ rest interval)	EXs	↑ ELV-miRNAs (miR1-3p, miR222-3p, miR23a-3p, miR208a-3p, and miR150-5p, miR486-5p and miR378a-5p)
									↓ ELV-miRNAs (miR16-5p)
									↔ ELV-miRNAs (miR499a-5p, miR24-2-5p, miR378b, miR494-3p, miR27a-5p, miR33a-3p)
Lovett et al. (2018)	*n* = 9 (M)	18–30	Two consecutive bouts	PMJ: 90% of maximal jump height		Plyometric exercises	PMJ: 10 sets x 10 jumps	EVs	↔ No ELVs size/number change 2h and 24 h post-Ex
				DHR: 10 km/h, 10% decline			DHR: 5 sets x 4′ of downhill running		↓ ELV-miRNA (miR-31)
Annibalini et al. (2019)	*n* = 8 (M) BMI: 24.4 ± (1.6)	23.7 ± (2.8)	Single bout	Maximal power (inertial resistance that yielded each participants’ peak power output)		Resistance exercise (FW + smith machine)	5 sets x 10 reps	EVs	↑ 2-fold ELV particles count post-ex
									↑ ELV-miRNAs (miR206, miR146a)
									↔ ELV-miRNAs (miR-16, miR-126, miR-133b)
Silver et al. (2020)	*n* = 20 (M/F) Male (*n* = 12) (BMI: 24.6 ± 3.1) Female (*n* = 8) (BMI: 24.5 ± 2.5)	M: 22.9 ± (2.6)	Single bout	70% of VO_2peak_	60′	Acute aerobic cycling exercise		EVs	↔ No ELV-miRNAs change post-Ex
		F: 23.0 ± (3.4)							No correlation between miRNA expression in ELVs and skeletal muscle
Just et al. (2020)	*n* = 9 (M) BMI: 23.3 ± (1.0)	21 ± (0.6)	Single bout	30% of 1RM		Acute partial-BFR resistance exercise (knee extensions)	5 sets x volitional failure	EVs	↔ No ELVs number/size change 1 h post-Ex
									↑ ELV-miRNAs (miR-182-5p, miR-1294, let-7b-5p, miR-451a, miR-16-5p, miR-36-3p)
									↓ ELV-miRNAs (miR-19b-3p, miR-17-5p, miR-221-3p, miR-150-5p, miR-340-5p, miR-21-5p)
Estébanez et al. (2021)	*n* = 50 (M/F) you*n*g group (*n* = 12) (BMI: 24.1 ± 1.5)	YG: 22.3 ± (2.1)	TG: 2x week/8 weeks	First four exercises: start at 40% of 1RM.		Resistance training	3 sets x 12-8-12 reps	EXs	↔ No ELVs number change
	Trai*n*i*n*g group (*n* = 28) (BMI: 27.4 ± 0.8)	TG: 72.6 ± (0.4)		Last four exercises: start at RPE 5 (OMNI-RES)			First four exercises: load increased by 5% each week		
	co*n*trol group (*n* = 10) (BMI: 28.9 ± 1.9)	CG: 73.6 ± (0.8)					Last four exercises: from 5 to 6 RPE in the first 4 weeks–from 6 to 8 in the last 4 weeks		
Garai et al. (2021)	*n* = 25 (M/F) you*n*g group (*n* = 14) (BMI: 21.6 ± 1.5)	YG: 23 ± (2.0)	YG: 3x week/6 mon	YG: RT (≤85% HRmax) AT (≤65% HRmax)	YG: 60′ x session	Resistance training + Aerobic Training		EXs	YG: 54 ELV-miRNAs altered post-ex
	Se*n*ior group (*n* = 11) (BMI: 27.9 ± 2.9)	SG: 62 ± (6.0)	SG: 54% daily basis, 46% at least 2x week for ≥25 years	SG: not assessed					YG/SG: ↓ ELV-miRNAs (let-7a-5p; let-7g-5p; miR-130a-3p; miR-142-3p; miR-150-5p; miR-15a-5p; miR-15b-5p; miR-199a-3p; miR-199b-3p; miR-223-3p; miR-23a-3p, and miR-451a-3p)
									SG: ↑ miR-144-3p; ↓ miR-411-5p
Da Silva et al. (2021)	*n* = 8 (M) experime*n*tal group (*n* = 4) co*n*trol group (*n* = 4)	EX: 66.2 ± (12.9)	3x week/8 weeks	Warm-up: comfortable rate	30′ x session	Aerobic Interval cycling training			↑ miR-106a-5p, miR-103a-3p, and miR-29a-3p
		**CG:** 63.5 ± (9.6)		Training: 15″ at 80% HR_6MWT_/45″ at 55%–60% HR_6MWT_					
				Cooling-off: comfortable rate					
Maggio et al. (2023)	*n* = 19 (M) AAE/AT/AMAE (*n* = 13) (BMI: 22 ± 3.2) AAT (*n* = 6) (BMI: 20.6 ± 2.8)	AAE/AT/AMAE: 20.1 ± (0.6)	AT: 3x week x8 weeks	AT: 55% of their VO_2max_	AT: 40′ x session	AT: Aerobic exercise		EVs	AT:
		AAT: 23.3 ± (6.8)	AAE: single bout	AAE:/	AAE:/	AAE: Steady-state aerobic exercise			↔ No ELVs number change
			AMAE: single bout	AMAE: personalized initial speed, increment of each 1-min stage calculated using a standard procedure, aiming to allow the attainment of the ˙VO_2max_ within 10′, constant grade of 1%	AMAE**:/**	AMAE: treadmill graded exercise test			↔ ELV-miRNAs (miR-133b, miR-206)
			AAT: 23 sessions within 15 days	AAT:/	AAT:**/**	AAT: Altitude (2,000 m a.s.l.) aerobic exercises			↑ ELV-miR-146a
									AAE:
									↔ No ELVs number change
									↑ ELV-miRNAs (miR-133b, miR-206, and miR-146a)
									AMAE**:** ↔ No ELVs number change
									↑ ELV-miRNAs (miR-206, miR-133b, miR-486-5p, miR-181a-5p, miR-16)
									**AAT:** ↔ No ELVs number change
									↔ ELV-miRNAs

CyG, cycling group; TmG. treadmill group; ↑, increase; ↓, decrease; ↔, No pre/post change; AAE, acute aerobic exercise; PMJ, plyometric jump; DHR, downhill running; FW, flywheel; BFR, blood flow restriction; YG, young group; TG, training Group; CG, control group; SG, senior group; RT: resistance training; AT: aerobic training; EX: experimental group; AMAE: acute maximal aerobic exercise; AAT, altitude aerobic training; a.s.l., above Sea Level; EVs, extracellular vesicles; EXs, exosomes; ELVs, exosome-like-extracellular vesicles; miRNAs, microRNAs.

## 3 Exercise and exosome-like extracellular vesicles

ELVs have been shown to function as signal transducers that are secreted into the blood following PA. These ELVs connect with target cells through surface interaction or membrane fusion, which could mediate the release of the ELVs contents into the target cells and initiate downstream signalling (Safdar and Tarnopolsky, 2018).

Exercise-induced ELVs may improve cognitive performance indirectly by transporting molecules that stimulate neurogenesis in the hippocampus. Irisin and cathepsin B (CTSB) are both myokines released from EVLs during exercise (Safdar and Tarnopolsky, 2018) that have been shown to improve memory and induce adult neurogenesis in the hippocampus via induction of brain-derived neurotrophic factor (BDNF) (Moon et al., 2016), proving to be neuroprotective in AD patients by reducing β-Amyloid Peptide (the main component of amyloid plaques) levels, although this remains controversial (Morris et al., 2014; Selkoe and Hardy, 2016; Chen et al., 2017).

Only recently, it has been recognized that physical exercise has substantial benefits on executive and cognitive functions, influenced by exercise parameters. Furthermore, even acute exercise plays a vital role in priming cellular pathways, leading to structural and functional adaptations observed in regular exercisers. Although both aerobic and resistance exercise, for instance, acutely increases BDNF values, aerobic exercise shows greater variation. The effects of physical exercise on circulating BDNF values found in subjects with NDs seem to be comparable to those exerted on the general population (Lippi et al., 2020).

Many studies have shown that different types of training can modulate ELVs in different ways, for example, a single bout of exhaustive aerobic exercise triggers the release of EVLs, which are cleared from the circulation during the early recovery period after cycling but remain elevated after running exercise (Frühbeis et al., 2015). A few molecules from the periphery can cross the blood-brain barrier (BBB) and interact with the brain, including ELVs that can migrate through the BBB via the transcellular pathway (Chen et al., 2016). Pharmacological studies, aimed at the treatment of brain diseases such as PD (Haney et al., 2015), have exploited this inherent ability of the ELVs to deliver drugs into the brain, or simply confirmed how these can actually cross the BBB (Chen et al., 2016) or carry a genetic cargo such as small-interfering RNA (siRNA), small RNA molecules capable of inhibiting the expression of a given gene to prevent the production of harmful proteins (Alvarez-Erviti et al., 2011). The ability of ELVs to transport myokines released during exercise and their ability to penetrate the BBB, allows us to speculate that ELVs may play an important role in muscle-brain crosstalk and, as a result, in NDs.

Different studies suggest that ELVs are released by skeletal muscle (SkM) cells and can be found in the bloodstream. Guescini et al. (2015) found that muscle tissue releases ELVs carrying miRNAs into the bloodstream in response to physical exercise, similar findings were observed by Rome et al. (2019) and Trovato et al. (2019). Increased plasma levels of myokines and proteins highly enriched in ELVs were observed in humans after an hour of cycle exercise (Whitham et al., 2018), and ELVs packed with hundreds of peptides were also discovered to be released from skeletal muscles following running exercise (Frühbeis et al., 2015). Estrada et al. (2022), using targeted immunocapture of tetraspanin-expressing particles (a method for isolating specific ELVs subpopulations based on the presence of tetraspanin proteins), found that SkM myofiber ELVs account for 4%–5% of circulating ELVs under free-living conditions, confirming that SkM-ELVs, while accounting for a smaller percentage of all ELVs produced, can reach circulation *in vivo* and their potential contribution to systemic physiological functions in distant cells and tissues. Overall, this evidence suggests that ELVs produced by SkM cells are released into the bloodstream and may play a role in muscle communication and homeostasis. Additionally, up to 75% of reported myokines were found to exist in ELVs (Safdar et al., 2016), highlighting the possibility that ELVs mediate exercise-induced myokine release.

However, a recent study (Watanabe et al., 2022), exploiting a proteomic approach used to identify potential tissue-specific ELVs marker proteins (i.e., ATP2A1, β-enolase, and desmin) that characterize ELVs derived from SkM, showed limited presence of SkM-ELVs in blood circulation, similar to Ismaeel et al. (2023), and they were hardly detected in the plasma, even after exercises, suggesting that SkM-ELVs do not appear to constitute a significant proportion of circulating ELVs, instead they were found to be highly accumulated within the SkM interstitium, indicating that they predominantly function within the tissue microenvironment. Furthermore, Watanabe et al. (2022) highlight that exercise-induced ELVs are more likely to be derived from leukocytes, platelets, and endothelial cells, while not ruling out the possibility that small but significant amounts of SkM-ELVs may enter the bloodstream and participate in inter-organ communication, as previously observed (Castano et al., 2022; Estrada et al., 2022).

These conflicting results may be due, at least in part, to the absence of specific SkM-ELVs markers defined for *in vivo* analysis, but they highlight the necessity of further research and, consequently, the current results should be viewed with caution.

## 4 How FITT-VP parameters modulate ELVs and microRNAs cargo

The potential benefits produced by exercise through EVLs are mainly due to their cargo. It is known that these vesicles can transport a variety of molecules including proteins, lipids, DNA, messenger RNAs (mRNA), siRNA and microRNA (miRNA); the latter has recently been the focus of different research (Karvinen et al., 2020; Doncheva et al., 2022; Garcia-Martin et al., 2022; Xu et al., 2022), showing how some miRNAs are taken into EVLs and released into the bloodstream without being degraded. Furthermore, these circulating miRNAs (c-miRNAs) can move to other cells and modulate their functions contributing to the pathogenesis of different diseases (Valadi et al., 2007).

miRNAs are small, single-stranded organized (∼19–22 nucleotide) sequences of RNAs that do not code for any protein but are functional in post-transcriptional regulation processes of coding genes and intercellular communication (Mohr and Mott, 2015). They primarily function by attaching to complementary mRNA sequences in recipient cells, interfering with the translational process, and stopping or changing the production of proteins.

Since miRNA species are known to play significant roles in a wide range of physiological and pathological processes and their dysregulation was observed in several NDs likewise ALS (Tasca et al., 2016; Pegoraro et al., 2017), AD, HD, and PD (Rajgor, 2018; Roser et al., 2018), they may also be engaged in the health benefits of exercise in terms of disease prevention; therefore, understanding the role of miRNAs carried by ELVs, produced during exercise, and their downstream targets would help us better understand how preventative lifestyle actually functions at the cellular level (Garai et al., 2021).

There is still a debate regarding the association of miRNAs with ELVs. Some studies suggest that miRNAs are predominantly associated with RNA-binding proteins rather than being contained within ELVs (Hoy and Buck, 2012). Whereas other studies indicate that miRNAs can be protected and released within ELVs (Wang et al., 2022). Moreover, the functional role of ELV-contained miRNAs in recipient cells is still uncertain. Some studies have questioned their functionality due to the low abundance of miRNAs in ELVs and the difficulty in evaluating their impact on recipient cells. An *in vitro* study (Albanese et al., 2021) found that, while ELVs showed interaction with various target cells, the fusion and transfer of ELV cargo into the cytoplasm of recipient cells were not detected, suggesting limited functional transfer of ELV-contained miRNAs. Conversely, other studies have reported the functional effects of miRNAs packed into the ELVs, suggesting that vesicular miRNAs can be internalized by a target cell regulating its gene expression (Rome et al., 2019; Castano et al., 2022).

However, altered levels of miRNAs are reported for numerous NDs and particularly type of miRNAs (miRs), defined as gatekeepers of both the nervous and immune system, seem capable of simultaneously modulating immune cell activation and neuronal function (Juźwik et al., 2019); interestingly miRNA expression changes after exercise (Guescini et al., 2015; D’Souza et al., 2018).

Guescini et al. (2015) reported that after acute aerobic exercise (40 min, vigorous intensity 80% of VO_2max_, treadmill exercise) muscle-specific miRNAs (miR-181a-5p, miR-133b, and miR-206), carried by ELVs, appear significantly increased or show an increasing trend. Differently, Aoi et al. (2013) observe that after acute and chronic aerobic exercise (a single bout of steady-state cycling exercise at 70% VO_2max_ for 60 min cycling/exercise at 70% VO_2max_ for 30 min, 3 times/week for 4 weeks) no significant changes in specific muscle miRNAs are detected in serum, except for miR-486 whose higher levels were observed in the early stage of HD (Packer et al., 2008; Karnati et al., 2015), which appears significantly decreased. After two consecutive bouts of mild muscle-damaging acute exercise (10 sets of 10 plyometric jumps at 90% of their maximum achievable jump height, with a 1 min interval between sets, 5 min rest followed by 5 sets of 4 min bouts of downhill running at a speed of 10 km/h, 10% decline, and 2 min standing interval between sets) the miR-31 cargo of circulating EVLs significantly decreased by 24 h post-performance of mild to moderate intensity (Lovett et al., 2018). Similarly, after a single bout of high-intensity interval exercise, was found that the increase in EVLs release returned to resting level after recovery, while the identification of 12 miRNAs as significantly altered in muscle and ELVs but not in plasma emphasizes the uniqueness of the signature of ELVs miRNAs as an independent component that may be an important mediator of cross-talk between tissues throughout the body, following exercise training (D’Souza et al., 2018).

Annibalini et al. (2019) report that after a single acute iso-inertial resistance exercise at the flywheel (5 sets of 10 maximal squats with 3 min rest between sets), there was an acute increase in the levels of circulating ELVs and the muscle-specific miRNAs (miR-206 and miR-146a) carried by them, that could be involved into the inflammatory response (Baggish et al., 2011). Some miRNAs, including miR-146a, are found to be involved in the regulation of inflammatory processes and are upregulated in NDs as AD (Lukiw et al., 2008), but the evidence is still conflicting (Ammal Kaidery et al., 2021). However, brain inflammation appears to have a dual function: to protect the brain from pathogens and neurotoxic agents during an acute phase response by promoting tissue repair (Yong et al., 2019), but to become detrimental when a chronic response sets in (Kinney et al., 2018).

In contrast, similarly to Lovett et al. (2018), another study (Estébanez et al., 2021) does not observe changes in plasma levels of ELVs and miRNAs (miR-146a), after 8 weeks of mild to moderate intensity resistance training (2 sessions/week, 3 sets/12-8-12 repetitions) in elderly subjects. Similarly, Just et al. (2020) after a single bout of low-intensity ischemic resistance exercise (BFRE) (5 sets of knee extension to volitional failure at 30% of 1RM) do not observe an increase in muscle-specific miRNAs packed into the ELVs and, likewise, Silver et al. (2020) did not observe significant modifications in the expression of miRNAs (miR-1, miR-16, miR-23b and miR-133a/b) carried by ELVs after a single acute bout of endurance exercise (cycling for 60 min at 70% of VO_2peak_).

Maggio et al. (2023) studied how miRNAs carried in ELVs are modulated following different protocols of aerobic exercises: acute aerobic activity (AAE) (single steady-state moderate-intensity workout on a treadmill) and aerobic training (AT) (moderate to vigorous intensity 40 min, 3 days/week for 8 weeks, 55% of their VO_2max_), maximal aerobic exercise (AMAE) (graded exercise test to exhaustion on a treadmill) and 2 weeks of high altitude aerobic training (AAT) (15 days of training camp at 2,000 m altitude, 23 sessions). After AAE they observe only an increasing trend in EVLs, but no significant upregulation of miRNAs carried by ELVs except for miR-146a; in contrast, these stimuli do not appear sufficient to generate a similar response, after AT. Following AMAE no change in the number of ELVs is detected, while there is an immediate post-exercise increase in miRNAs (miR-206, miR-133b, miR-486-5p, miR-181a-5p, miR-16) carried by ELVs that return to basal levels faster than at AAE, suggesting that it may be related to exercise intensity. Finally, no increase in the number of ELVs is observed following AAT, but rather a decrease, with no significant change in miRNA expression levels.

Few studies have investigated the effects of exercise on the modulation of ELVs and the miRNAs they contain and carry, over the long term. Garai et al. (2021) do so by evaluating the effects of a concomitant training protocol (60 min, 3 times/week of endurance and strength training) performed for 6 months, on the expression of specific ELV-transported miRNAs, in previously sedentary healthy young adults (YG) and comparing this profile with that present in healthy senior subjects who have been engaged in regular exercise (endurance and strength training) for at least 25 years (SG). They thus identified 12 similarly regulated miRNAs in both the YG and SG groups. They observe a reduction in some specific miRNAs (miR-23a, miR-451a, miR-223-3p, miR-150-5p, miR-15a, miR-142-3p) among which the expression of miR-142-3p was found to be significantly reduced compared to sedentary subjects.

While the expression levels of these miRNAs, whether carried by ELVs or present freely in the bloodstream, have been observed to be significantly higher in the serum of individuals affected by various NDs (Barbagallo et al., 2020; Da Silva et al., 2021), the current evidence does not conclusively establish a causal relationship between regular physical activity and the reduction of the risk of developing neurodegenerative diseases or the improvement of their symptoms through the modulation of ELVs and specific miRNAs carried by them. These, however, may provide a non-negligible biological plausibility in support of a possible role played by exercise and may allow hypotheses to be generated about which clusters of integrated pathways may be involved. Further studies are needed to move beyond speculative inferences and establish a link of causality.

## 5 Limitations and future directions

Although this area of research is promising, several limitations arise in structuring studies. The first of these is the difficulty of studying different EVs subpopulations; assigning an EV to a particular biogenesis pathway is incredibly difficult unless, for example, the EV is captured in the act of release with live imaging techniques. This is because a consensus on specific markers of EV subtypes, such as endosomal-derived “exosomes” and “ectosomes” (microparticles/microvesicles) derived from the plasma membrane, has not yet developed (Théry et al., 2018).

Furthermore, although it can be considered a marker of the onset and progression of some NDs, challenges and limitations are emerging in understanding the functional role of miRNAs contained within ELVs and their impact on recipient cells.

Despite growing evidence, there are still few studies about the effects exerted by exercise on ELVs and their cargo, even fewer that specifically assess FITT-VP parameters and have been conducted on subjects with NDs. Even in the latter case, the studies are limited by the absence of control of dosages and types of treatments and by only man samples (Da Silva et al., 2021). Furthermore, in some works, training parameters such as volume and progression are not always controlled and almost all studies use small samples.

Finally, it should be considered that many studies perform assessments immediately after a single exercise, focusing on the acute and transient aspects of exercise, however serum-level changes in the number and content of ELVs may be influenced by stress-related factors (Beninson and Fleshner, 2014; Hou et al., 2019), while only a few studies analyze long-term effects (Aoi et al., 2013; Estébanez et al., 2021; Garai et al., 2021; Da Silva et al., 2021; Maggio et al., 2023).

New studies should focus on larger samples of subjects affected or at risk of developing NDs, evaluating the long-term effects of exercise on modulation of ELVs and their cargo, with specific attention to the packed miRNAs in ELVs as several evidences support their ability to be assimilated by target tissue cells, contrary to free miRNAs, and regulate gene expression (Valadi et al., 2007; Rome et al., 2019; Xu et al., 2022; Liu and Wang, 2023). Special attention should also be paid to controlling the FITT-VP parameters of the intervention protocol, as these aspects could positively or negatively affect the outcomes.

## 6 Conclusion

Exercise has been associated with potential benefits in terms of neuroprotection and the prevention of NDs. The effects of exercise on ELVs and their cargo, specifically miRNAs, may be one of the mechanisms underlying these observed benefits. Different exercise parameters can lead to distinct changes in ELVs levels and miRNAs expression profiles; however, given the variety of exercise protocols, the optimal dose-response connection of exercise, and related FITT-VP parameters, remain uncertain.

It seems that acute aerobic exercise practised at vigorous intensity, can result in increased levels of ELVs and muscle-specific miRNAs (miR-181a-5p and miR-133b) carried by EVLs. This suggests that acute aerobic exercise may have an impact on miRNA expression in EVLs, differently from endurance training and chronic exercise. Acute resistance exercise at mild to moderate intensity, differently from low-intensity ischemic resistance exercise, can induce acute increases in ELVs levels and muscle-specific miRNAs (miR-206 and miR-146a) that may be involved in the inflammatory response, although the evidence for their role in inflammation and NDs is still conflicting. Long-term concomitant exercise training (endurance/strength) can result in the downregulation of specific ELV-transported miRNAs (miR-142-3p, miR-23a) potentially contributing to the reduction of the risk of developing NDs.

Overall, exercise parameters have been shown to influence the release of ELVs and the modulation of miRNAs carried by these vesicles. Further research is needed to fully understand the specific mechanisms and implications of exercise-induced ELVs release and miRNAs modulation in the NDs context, as well as their potential therapeutic applications.
